# Expression of cyanobacterial genes enhanced CO_2_ assimilation and biomass production in transgenic *Arabidopsis thaliana*

**DOI:** 10.7717/peerj.11860

**Published:** 2021-08-09

**Authors:** Anum Zeb Abbasi, Misbah Bilal, Ghazal Khurshid, Charilaos Yiotis, Iftikhar Zeb, Jamshaid Hussain, Ayesha Baig, Mohammad Maroof Shah, Safee Ullah Chaudhary, Bruce Osborne, Raza Ahmad

**Affiliations:** 1 Department of Biotechnology, COMSATS University Islamabad, Abbottabad Campus, Abbottabad, KP, Pakistan; 2 School of Biology and Environmental Sciences, University College Dublin, Belfield, Dublin, Ireland; 3 Department of Biological Applications and Technology, University of Ioannina, Ioannina, Greece; 4 Department of Biology, School of Science and Engineering, Lahore University of Management Sciences, Lahore, Punjab, Pakistan

**Keywords:** CO_2_ assimilation, Photosynthesis, Photorespiration, Cyanobacterial genes, *Arabidopsis thaliana*, Biomass

## Abstract

**Background:**

Photosynthesis is a key process in plants that is compromised by the oxygenase activity of Rubisco, which leads to the production of toxic compound phosphoglycolate that is catabolized by photorespiratory pathway. Transformation of plants with photorespiratory bypasses have been shown to reduce photorespiration and enhance plant biomass. Interestingly, engineering of a single gene from such photorespiratory bypasses has also improved photosynthesis and plant productivity. Although single gene transformations may not completely reduce photorespiration, increases in plant biomass accumulation have still been observed indicating an alternative role in regulating different metabolic processes. Therefore, the current study was aimed at evaluating the underlying mechanism (s) associated with the effects of introducing a single cyanobacterial glycolate decarboxylation pathway gene on photosynthesis and plant performance.

**Methods:**

Transgenic *Arabidopsis thaliana* plants (GD, HD, OX) expressing independently cyanobacterial decarboxylation pathway genes *i.e*., glycolate dehydrogenase, hydroxyacid dehydrogenase, and oxalate decarboxylase, respectively, were utilized. Photosynthetic, fluorescence related, and growth parameters were analyzed. Additionally, transcriptomic analysis of GD transgenic plants was also performed.

**Results:**

The GD plants exhibited a significant increase (16%) in net photosynthesis rate while both HD and OX plants showed a non-significant (11%) increase as compared to wild type plants (WT). The stomatal conductance was significantly higher (24%) in GD and HD plants than the WT plants. The quantum efficiencies of photosystem II, carbon dioxide assimilation and the chlorophyll fluorescence-based photosynthetic electron transport rate were also higher than WT plants. The OX plants displayed significant reductions in the rate of photorespiration relative to gross photosynthesis and increase in the ratio of the photosynthetic electron flow attributable to carboxylation reactions over that attributable to oxygenation reactions. GD, HD and OX plants accumulated significantly higher biomass and seed weight. Soluble sugars were significantly increased in GD and HD plants, while the starch levels were higher in all transgenic plants. The transcriptomic analysis of GD plants revealed 650 up-regulated genes mainly related to photosynthesis, photorespiratory pathway, sucrose metabolism, chlorophyll biosynthesis and glutathione metabolism.

**Conclusion:**

This study revealed the potential of introduced cyanobacterial pathway genes to enhance photosynthetic and growth-related parameters. The upregulation of genes related to different pathways provided evidence of the underlying mechanisms involved particularly in GD plants. However, transcriptomic profiling of HD and OX plants can further help to identify other potential mechanisms involved in improved plant productivity.

## Introduction

Atmospheric CO_2_ (C_a_) concentration has increased due to human activities from 280 ppm in preindustrial times to 400 ppm today (Thompson et al., 2017) and this is considered the major driver of climatic changes (Jovanović et al., 2015). Associated increases in global temperature and reductions in soil water availability are associated with rising C_a_, posing significant threats to ecosystem sustainability and crop productivity (Sperry et al., 2019). Plants are the natural carbon sequesters and rely on photosynthesis to sequester and store large quantities of C_a_ in the form of living plant biomass, both above and belowground (Prior et al., 2011; Sperry et al., 2019).

During photosynthesis, ribulose-1,5-bisphosphate-carboxylase/oxygenase (Rubisco) fixes atmospheric CO_2_ (Parry et al., 2013). Rubisco is a bifunctional enzyme which can add CO_2_ (carboxylation) as well as O_2_ (oxygenation) to the substrate ribulose-1,5-bisphosphate (RuBP) (Janssen et al., 2014). The carboxylation reaction yields two molecules of phosphoglycerate (3-PGA) which is then reduced to sugar molecules in the Calvin Benson cycle (CBC) (Carmo-Silva et al., 2015). However, the oxygenation reaction results in the production of one molecule each of 3-PGA and phosphoglycolate (2-PG) (Carmo-Silva et al., 2015). 2-PG and its immediate product glycolate (GCA) are toxic molecules and need to be metabolized into 3-PGA (Maurino & Peterhansel, 2010). Plants employ photorespiration to catabolize 2-PG and GCA through series of enzymatic reactions that are distributed over chloroplast, peroxisome, and mitochondrion (Hagemann & Bauwe, 2016). Moreover, during photorespiration, hydrogen peroxide (H_2_O_2_) and ammonia (NH_3_) are produced, along with the release of one molecule of already fixed CO_2_ (Maurino & Peterhansel, 2010). Photorespiration considerably influences crop productivity by reducing yields in C_3_ crops by as much as 50% under severe conditions (Basler et al., 2016; South et al., 2019).

In a rising CO_2_ and increasing global temperature scenario in combination with increased abiotic stresses such as salinity and drought, photosynthesis becomes constrained due to decreased stomatal conductance (g_s_) which limits CO_2_ supply, resulting in an increase in photorespiration (Maiti & Satya, 2014; South et al., 2019). However, under these conditions, photorespiration may have some benefits, by dissipating energy generated during the light reactions and regenerating oxidized nicotinamide adenine dinucleotide (NADP) (Betti et al., 2016). Despite the positive role of photorespiration when photosynthesis is limited by abiotic stresses-mediated enhanced photorespiration this leads to the overproduction of reactive oxygen species (ROS) (Bose, Rodrigo-Moreno & Shabala, 2014; Sharma et al., 2019; Yadav et al., 2020). Interestingly, salinity can lead to the onset of a multitude of responses including osmotic effects, sodium toxicity, and enzyme denaturation, which can further hamper plant metabolic process (Munns & Tester, 2008; Carillo et al., 2011; Negrão, Schmöckel & Tester, 2017). In addition to this, salinity can also inhibit the translocation of photosynthate in plants leading to accumulation of sugars in leaves which may repress the genes encoding the Rubsico enzyme (Moghaieb et al., 2011).

Plants being natural carbon sequesters, can be modified through genetic engineering to enhance CO_2_ assimilation and reduce photorespiratory losses (Ruan, Shao & Teixeira Da Silva, 2012). Overexpression of glycolate oxidase (GO), a photorespiratory pathway gene, in rice has been reported to mitigate photorespiration and improve photosynthesis under stress conditions (Cui et al., 2016). Reports on synthetic metabolic pathways that bypass photorespiration, but do not completely eliminate it, have been shown to reduce photorespiratory carbon loss and thereby enhance photosynthetic efficiency and biomass yield (Kebeish et al., 2007; Maier et al., 2012; Dalal et al., 2015; South et al., 2019). In this context, complete photorespiratory bypasses have been engineered in plants by Kebeish et al. (2007), Maier et al. (2012), Dalal et al. (2015), Shen et al. (2019), and South et al. (2019), all of which resulted in an increased plant biomass (Kebeish et al., 2007; Maier et al., 2012; Dalal et al., 2015; Shen et al., 2019; South et al., 2019). Interestingly, engineering of individual genes from these engineered photorespiratory bypasses have also resulted in an enhancement in photosynthetic efficiency (Kebeish et al., 2007; Nölke et al., 2014; Dalal et al., 2015; Ahmad et al., 2016). The premier study on photorespiratory bypasses was reported by Kebeish et al. (2007) in which three enzymes *i.e*. glycolate dehydrogenase (GDH) comprising of three subunits (D, E and F), glyoxylate carboligase (GCL) and tartronic semialdehyde reductase (TSR) from the bacterium *E. coli* were utilized to catabolize GCA in the chloroplast. The transgenic *Arabidopsis thaliana* showed enhanced growth and photosynthetic rate (Kebeish et al., 2007). In addition to the use of a complete bypass, Kebeish et al. (2007) also reported an enhancement in photosynthesis and biomass accumulation by engineering only GDH in *Arabidopsis thaliana* (Kebeish et al., 2007). Later on, Nölke et al. (2014) also reported an increase in potato yield by engineering GDH alone. Engineering of single gene in these studies has also improved biomass accumulation, however, the exact mechanism behind the increase in biomass is not very much clear. Subsequently, Dalal et al. (2015) utilized only GDH to improve net photosynthesis in the biofuel crop *Camelina Sativa* (Dalal et al., 2015). The transgenic plants with a single gene substitution exhibited enhanced photosynthesis and biomass accumulation. To elucidate the underlying mechanism associated with the single gene modification, Dalal et al. (2015) also carried out transcriptomic profiling. Transcriptomic analysis revealed that the GDH expressing lines showed upregulation in genes related to sugar transporters, plastidic fatty acid biosynthesis, calcium transporting ATPases, and auxin response proteins (Dalal et al., 2015). This upregulation in different genes suggested an indirect role for the single gene transformation in regulating the plant transcriptome, which improved the biomass accumulation (Dalal et al., 2015).

**Figure 1:**
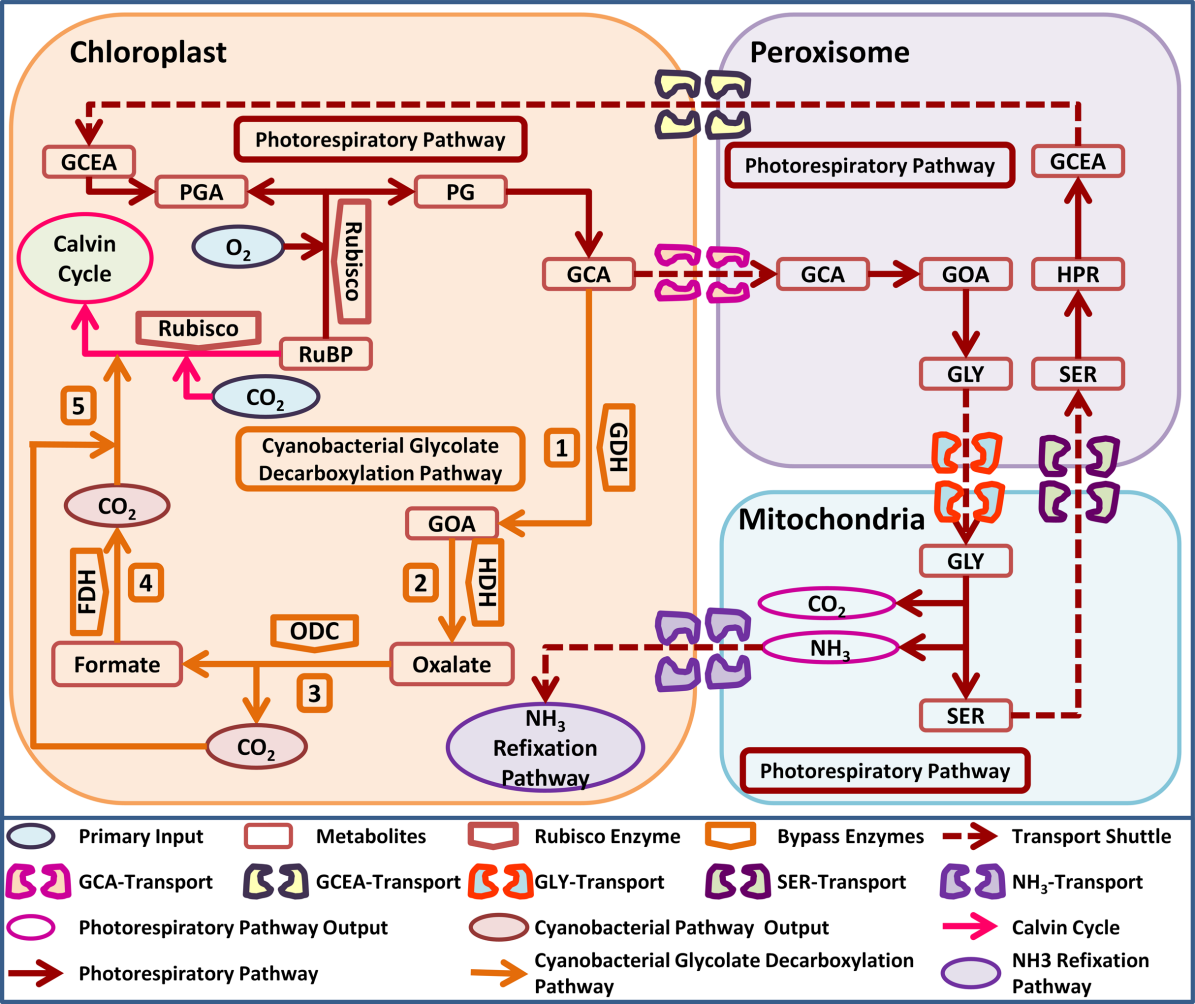
A schematic representation of plant native photorespiratory and cyanobacterial glycolate decarboxylation pathway genes transformed independently into *Arabidopsis thaliana*. Atmospheric CO_2_ and ****Oxygen (O_2_) combines with metabolite ribulose 1, 5 bisphosphate (RuBP) in a Rubisco catalyzed reaction. The carboxylation reaction initiates Calvin cycle while oxygenation reaction yields phosphoglycerate (3-PGA) and phosphoglycolate (2-PG). Catabolism of 2-PG onset the photorespiratory pathway which is distributed over three compartments *i.e*. chloroplast, peroxisome, and mitochondria, respectively. 2-PG is converted into glycolate (GCA), glyoxylate (GOA), glycine (GLY), serine (SER), hydroxypyruvate (HPR), glycerate (GCEA), and 3-PGA, respectively. The photorespiratory pathway outputs *i.e*. CO_2_ and ammonia (NH_3_) have also been added in the mitochondria. Cyanobacterial glycolate decarboxylation pathway (labelled 1–5) genes integrated in chloroplast at GCA level. The cyanobacterial pathway comprises of glycolate dehydrogenase (GDH) which produces GOA from GCA, hydroxyacid dehydrogenase (HDH) yields oxalate, oxalate decarboxylase produces formate and one molecule of CO_2_, and formate dehydrogenase yields another molecule of CO_2_. These two molecules of CO_2_ produced after the catabolism of GCA in chloroplast depicts the photorespiratory bypass output and are utilized in Rubisco catalyzed carboxylation reaction.

Interestingly, cyanobacteria also possess three photorespiratory pathways *i.e*. plant like (photorespiratory pathway), *E. coli*-like glycerate and a unique glycolate decarboxylation pathway (Eisenhut et al., 2008). The cyanobacterial glycolate decarboxylation pathway decarboxylates GCA into glyoxylate (GOA) through the activity of GDH, which is then converted into oxalate through hydroxyacid dehydrogenase (HDH). Oxalate is catabolized by oxalate decarboxylase (ODC) into formate and one molecule of CO_2_ is released, and lastly, a second molecule of CO_2_ is released by the catabolism of formate through formate dehydrogenase (FDH) (Fig. 1) (Eisenhut et al., 2008). Interestingly, the cyanobacterial glycolate decarboxylation pathway yields two molecules of CO_2_ during GCA catabolism and offers an opportunity to utilize it in plants to improve photosynthesis by reducing photorespiration. The released CO_2_ molecules from the decarboxylation pathway can increase the CO_2_ concentration in the vicinity of Rubisco, which can improve photosynthesis.

Recently, we have reported transgenic *Arabidopsis thaliana* plants independently expressing novel cyanobacterial decarboxylation pathway genes *i.e*. GDH, HDH, and ODC (GD, HD, and OX transgenic plants) (Bilal et al., 2019). The resultant transgenic plants showed improved growth and chlorophyll content (Bilal et al., 2019). These positive results led us to further analyze these transgenic plants to elucidate the underlying mechanism of single gene transformations in regulating photosynthesis, as well as other plant metabolic processes, which can improve plant growth and biomass accumulation. To investigate this, the photosynthetic and growth-related parameters of transgenic plants *i.e*. GD, HD, and OX, were evaluated. Furthermore, transcriptomic profiling of the GD transgenic plants was carried out to provide an insight into the effect of a single gene transformation on the transcriptome.

These results prompted us to further analyze several photosynthetic parameters, along with seed and plant biomass accumulation and seed germination under salt stress mediated photorespiratory conditions in GDH, HDH, and ODC expressing transgenic plants. The transgenic plants showed variable but general enhancements in gas exchange and fluorescence-related assessments of photosystem II activity. Interestingly, the OX plants also displayed a significant reduction in their relative photorespiration rate. Alongside this, an improvement was also observed in seed and plant biomass as compared to wild type (WT) plants. Moreover, soluble sugars and starch level was also observed to increase in transgenic lines as compared to WT. These results highlight the potential of engineering individual cyanobacterial decarboxylation photorespiratory pathway genes in C_3_ plants that can enhance CO_2_ assimilation and crop productivity.

## Material and Methods

### Plant material and growth conditions

The cyanobacterial glycolate decarboxylation pathway genes *i.e*. glycolate dehydrogenase (GDH; Accession No. sll0404), hydroxyacid dehydrogenase (HDH; Accession No. slr1556), and oxalate decarboxylase (ODC; Accession No. sll1358) in cyanobacteria *Synechocystis* sp. strain PCC 6803 as reported by Eisenhut et al. (2008) were selected to construct a plant expression vector. The expression vectors expressing independently the cyanobacterial GDH, HDH, and ODC under the control of cauliflower mosaic virus 35S promoter were utilized for the transformation of *Arabidopsis thaliana* plants. The genes were translationally targeted to the chloroplast. Single copy homozygous *Arabidopsis thaliana* plants expressing cyanobacterial GDH, HDH, and ODC were then developed and evaluated for plant growth parameters (Bilal et al., 2019). In this study, seeds from the same batch developed by Bilal et al. (2019) were further utilized and grown in pots (4 inches) comprising a 2:1 ratio of peat moss and perlite. In the previous study, different lines expressing GDH did not show major difference in the measured traits. A similar trend was observed in different lines expressing HDH, and ODC as well. Therefore, we utilized biological replicates of GD, HD, and OX plants in the current study. For the determination of gas exchange, chlorophyll florescence and biomass related parameters six biological explicates originating from the same experiment were used. Moreover, it should be noted that seeds from the same batch of the plants were used to perform all the experiments in this study. Plants were grown at 22–25 °C and a 16/8 h day night photoperiod and utilized to evaluate the effect of single cyanobacterial gene on different photosynthetic and plant growth related parameters.

### Gas exchange and chlorophyll florescence analysis

The gas exchange and chlorophyll fluorescence characteristics of the wild type (WT) and transgenic plants were measured in a ventilated room with a Li-cor 6400 gas analyzer (Li-cor, Lincoln, Ne, USA) fitted with a 6400-40 Leaf Chamber Fluorometer. The 6400-40 Leaf Chamber Fluorometer is equipped with red and blue light LEDs, thus functioning as both a fluorometer and a light source for the measurements. Intact, fully expanded leaves of 8-week-old plants were used for the measurements. The leaves were big enough to fully cover the 2 cm^2^ measurement window when they were clamped in the cuvette of the analyzer. The leaves were irradiated with an incident photon flux intensity (*I*) of 1,000 μmol m^−2^ s^−1^ until full photosynthetic induction, as judged from three consecutive stable readings of net photosynthetic rate (*A*), stomatal conductance (g_s_), and chlorophyll fluorescence-based photosynthetic electron transport rate (ETR). These three consecutive stable readings were used to calculate average values of *A*, g_s_, rate of transpiration (E), intercellular CO_2_ (C_i_), water use efficiency (WUE), light adapted quantum efficiency of photosystem II (ΦPSII) and ETR for each plant. Li-cor 6400 automatically calculates all the gas exchange parameters following the manufacturer’s equations. It also calculates Φ_PSII_ and ETR following the equations of Genty, Briantais & Baker (1989):

(1) $${{\Phi }_{{\rm PSII}}} = \displaystyle{{{\rm F}_{\rm m}^\prime - {{\rm F}_{\rm s}}} \over {{\rm F}_{\rm m}^\prime}}$$

(2) $${\rm ETR} = {{\Phi }_{{\rm PSII}}}fI{{\rm \alpha }_{{\rm leaf}}}$$

where ${{\rm F}_{\rm s}}$ is the steady-state fluorescence of the leaf in the light, ${\rm F}^{\prime}_{\rm m}$ is the maximum fluorescence value during a saturating light pulse, ${\rm f}$ is the fraction of absorbed light used by photosystem II (0.5), ${\rm I}$ is the photon flux density (μmol m^−2^ s^−1^) and ${{\rm \alpha }_{{\rm leaf}}}$ is the leaf absorptance assumed to be equal to 0.88 (Genty, Briantais & Baker, 1989).

The O_2_ and CO_2_ concentration in the cuvette, airflow, leaf temperature, and vapour pressure deficit (VPD) during the measurements were 21%, 400 ppm, 500 cm^3^ min^−1^, 21.4 ± 0.6 °C and 0.84 ± 0.07 kPa, respectively. The low VPD precluded stomatal closure and associated limitations on A.

After recording the light-adapted gas exchange and chlorophyll fluorescence parameters of the plants, the lights in the cuvette were switched off and the leaves were left to equilibrate in the dark for >5 min. Typically, the analyzer’s readings stabilized within 5–10 min. After that, and while leaf temperature was still controlled, three readings of dark respiration (R_d_) were recorded and used to calculate the average R_d_ for each plant. Subsequently, the quantum efficiency of CO_2_ assimilation of the plants was calculated using the previously recorded values of *A* as:

(3) $${{\Phi }_{{\rm C}{{\rm O}_{\rm 2}}}} = \displaystyle{{{\rm A} - {{\rm R}_{\rm l}}} \over {I{{\rm \alpha }_{{\rm leaf}}}}}$$

where R_l_ is the rate of respiration in the light assumed to be half of R_d_, an assumption commonly used to account for the suppression of respiration by the light.

### Estimation of photorespiration and photosynthetic electron transport rates attributable to carboxylation and oxygenation reactions

Prior to the gas exchange and chlorophyll fluorescence measurements at 21% O_2_ previously described, we used the same leaves to take combined photosynthesis and chlorophyll fluorescence measurements under non-photorespiratory conditions and variable light intensity. These measurements were subsequently used to calculate the rate of photorespiration (Pr) relative to gross photosynthesis (A_gross_) *i.e*. Pr/A_gross_ and the ratio of the photosynthetic electron flow attributable to carboxylation reactions over that attributable to oxygenation reactions (J_C_/J_O_) as described by Valentini et al. (1995). The combined measurements under non-photorespiratory conditions (*i.e*. 2% O_2_) were used to establish the linear relationships between Φ_PSII_, ${{\Phi }_{{\rm C}{{\rm O}_2}}}$ and the quantum efficiency of the photosynthetic linear electron flow (${{\Phi }_{{{\rm e}^ - }}}$), as described by Genty, Briantais & Baker (1989):

(4) $${{\Phi }_{{\rm PSII}}} = {\rm k}\cdot {{\Phi }_{{\rm C}{{\rm O}_2}}} + {\rm b} = 1/4\cdot {\rm k}\cdot {{\Phi }_{{{\rm e}^ - }}} + {\rm b}$$

where 4 is the number of electrons needed per CO_2_ molecule fixed.

According to the method, it can be assumed that these relationships hold under both photorespiratory and non-photorespiratory conditions and that the linear electron flow is uniquely devoted to the photosynthetic and photorespiratory processes. Following this and based on Eq. (4), ${{\Phi }_{{{\rm e}^ - }}}$ can be calculated as:

(5) $${{\Phi }_{{{\rm e}^ - }}} = 4\cdot \left( {{{\Phi }_{{\rm PSII}}} - {\rm b}} \right)/{\rm k}$$

and the total photosynthetic electron flow under photorespiratory conditions (J_T_) is:

(6) $${{\rm J}_{\rm T}} = I\cdot {{\rm \alpha }_{{\rm leaf}}}\cdot {{\Phi }_{{{\rm e}^ - }}}$$

and equal to:

(7) $${{\rm J}_{\rm T}} = {{\rm J}_{\rm C}} + {{\rm J}_{\rm O}}$$

where J_C_ and J_O_ are the photosynthetic electron flows attributable to carboxylation reactions over that attributable to oxygenation reactions, respectively.

Assuming the use of 4 electrons per CO_2_ molecule fixed and the release of one molecule of CO_2_ for every two oxygenated molecules of RuBP, J_C_ and J_O_ can be expressed as:

(8) $${{\rm J}_{\rm C}} = 4\cdot {A_{{\rm gross}}} = 4\cdot \left( {A + {{\rm R}_{\rm l}} + {\rm Pr}} \right)$$

(9) $${{\rm J}_{\rm O}} = 4\cdot \left( {2{\rm Pr}} \right)$$

Finally, from Eqs. (7), (8) and (9) J_C_, J_O_ and Pr can be calculated as:

(10) $${{\rm J}_{\rm C}} = 1/3\cdot \left[ {\; {{\rm J}_{\rm T}} + 8\cdot \left( {A + {{\rm R}_{\rm l}}} \right)} \right]$$

(11) $${{\rm J}_{\rm O}} = 2/3\cdot \left[ {\; {{\rm J}_{\rm T}} - 4\cdot \left( {A + {{\rm R}_{\rm l}}} \right)} \right]$$

(12) $${\rm Pr} = 1/12\cdot \left[ {{\rm \; }{{\rm J}_{\rm T}} - 4\cdot \left( {A + {{\rm R}_{\rm l}}} \right)} \right]$$

To calculate Pr, J_C_ and J_O_ and eventually Pr/A_gross_ and J_C_/J_O_, we used the *A* and R_l_ values measured with the gas exchange measurements at 21% O_2_ and the J_T_ estimated through the parallel chlorophyll fluorescence measurements under the same conditions (see previous section and Eqs. (5) and (6)).

The measurements under non-photorespiratory conditions were taken with the Li-cor 6400 gas analyzer. The cuvette conditions were the same as those reported in the previous section apart from the light intensity and O_2_ concentration. A compressed gas bottle containing a gas mixture composed of 98% N_2_ and 2% O_2_ was connected to the Li-cor 6400 air intake through a gas flow regulator, tubes and a T-piece. The T-piece was used to connect a 15 cm tube other than the two tubes connecting the gas bottle with Li-cor 6400, which functioned as an exhaust of the excess gas flow coming from the gas bottle. Upon clamping, the leaves were irradiated with an incident photon flux intensity (*I*) of 1,000 μmol m^−2^ s^−1^ until full photosynthetic induction. After that light intensity was stepwise decreased from 1,000 to 50 μmol m^−2^ s^−1^ (1,000, 750, 500, 300, 200, 100, 50). The readings of *A*, Φ_PSII_ and *I* at each step together with the estimates of R_l_ and ${{\rm \alpha }_{{\rm leaf}}}$ (see above) were subsequently used to establish the relationship between Φ_PSII_ and ${{\Phi }_{{\rm C}{{\rm O}_2}}}$ and estimate the ratios Pr/A_gross_ and J_C_/J_O_ as described above.

### Vegetative and seed biomass analysis

Above ground and root biomass of mature plants from each line including wild type was harvested and dried at 72 °C for 48 h. Dried plant material was weighed to determine vegetative dry weight. Silique length at physiological maturity was also measured using vernier caliper. Seeds were collected from siliques and the dry seed weight/plant was measured using an electronic balance.

### Quantification of soluble sugars and starch contents

The soluble sugars were quantified as described in Chow & Landhäusser (2004). Briefly, the sampling was performed at bolting stage and 8 h after illumination of light and shade dried leaves of WT and transgenic plants were utilized for the analysis. The extraction was performed with hot ethanol from 50 mg of powdered leaves. The ethanol mixed with powdered leaves were placed in water bath for 10 min at 95 °C. The extraction was performed three times and supernatant was used for sugars analysis. The absorbance for soluble sugars was recorded at 490 nm by using UV-visible spectrophotometer (HACH DR1900, USA). The pellet was utilized for quantification of starch as described by Chang (1979) and Lafont-Mandoza, Severiche-Sierra & Jaimes-Morales (2018). For this analysis, the crude starch leftover was dried at 50 °C to remove the ethanol. The pellet was dissolved in cold water followed by addition of iodine solution. This mixture was incubated at room temperature for 10 min and absorbance was recorded at 615 nm.

### Seed germination assay under salt stress conditions

To evaluate the performance of transgenic plants under salt stress, seeds from WT, GD, HD, and OX plants were sterilized initially with 70% ethanol for five minutes, and then the seeds were washed with sodium hypochlorite (NaOCl) for 10 min. Finally, seeds were washed with distilled water at least 5 times to remove salt particles and other impurities. Seeds were then grown in three different media *i.e*. (a) Murashige and Skoog (MS) media (Murashige & Skoog, 1962) supplemented with 1% sucrose (control), (b) MS media supplemented with 1% sucrose and 75 mM sodium chloride (NaCl), and (c) MS media supplemented with 1% sucrose and 100 mM NaCl. Sucrose was added to the media to avoid the NaCl shock to the germinating seeds of WT as well as transgenic plants. The sterilized seeds (50 each line, three replicates) were grown in petri plates and the plates were placed vertically in a growth chamber to ensure uniform germination. The growth conditions within growth chamber were set at 25 °C and 16/8 h light/dark condition. For 14 days seed germination data was collected at regular intervals and according to method described by Afsar et al. (2020) stress tolerance index was recorded (Afsar et al., 2020).

### RNA sequencing and differentially expressed genes analysis

For the transcriptome analysis, seeds (WT and GD) were taken from the same batch and plants were grown as described in the preceding section. Among the GD plants, GD16 was selected for transcriptomic analysis as representative of GD plants. Total RNA was extracted from *Arabidopsis thaliana* plants (WT and GD plants) grown in normal conditions described above. The leaf samples were collected at the start of bolting and after 6 hours of illumination. The quality of total RNA was checked by nanodrop (Nanodrop 2000; Thermo fisher, Waltham, MA, USA) and RNA of standard purity was utilized. The RNA integrity number (RIN) was determined by Agilent 2100 before proceeding further. Total amount of 2 µg of RNA (concentration ≥ 300 ng/µL) was utilized. The mRNA was purified and enriched by removing rRNA and DNA. The first strand cDNA was synthesized by using random N6 primer. Then the double stranded DNA was synthesized for subsequent processes. The sequencing was performed by MGI high-throughput sequencer. The raw data in FASTQ format was received which also contained corresponding sequencing quality. The raw data was filtered to remove the adapter information and clean data was generated for analysis of differential expressions. The MSA reference database was used to map the clean data by utilizing Bowtie2 software (Langmead & Salzberg, 2012). By using RSEM software gene expression profiling was conducted and it was normalized by FPKM (fragments per kilobases per million bases) (Li & Dewey, 2011). The differential expression of genes (DEGs) of GD plants in comparison of WT was performed by DESeq2 (Love, Huber & Anders, 2014). The log_2_ (fold change) ≥ 1 for up regulation and log_2_ (fold change) < 1 for down regulation was utilized, while the FDR was kept at <0.01. The enrichment analysis of GO and KEGG pathways was performed by using webserver for gene ontology (GO) *i.e*. agriGO 2.0 (Du et al., 2010; Tian et al., 2017) and KOBAS 3.0 (Wu et al., 2006; Xie et al., 2011). The enrichment analysis was performed utilizing corrected p values (*p < 0.05*) (Kong et al., 2015). The rich factor denotes the ratio of differentially expressed genes in a pathway term to the total genes annotated in the same pathway term (Kong et al., 2015). The transcriptomic sequence data was deposited to the National Center for Biotechnology Information (NCBI) under the accession SAMN18080037, which will be publicly available.

### Statistical analysis

The statistical analyses were carried out using Microsoft Excel 2010. The data were checked for normality and equal variances before we performed the student’s t-tests. The values were considered statistically significant at *P < 0.05*. The principal component analysis was carried out using MINITAB version 16.

## Results

### Independent expression of cyanobacterial genes enhanced photosynthetic efficiency of transgenic plants

**Figure 2:**
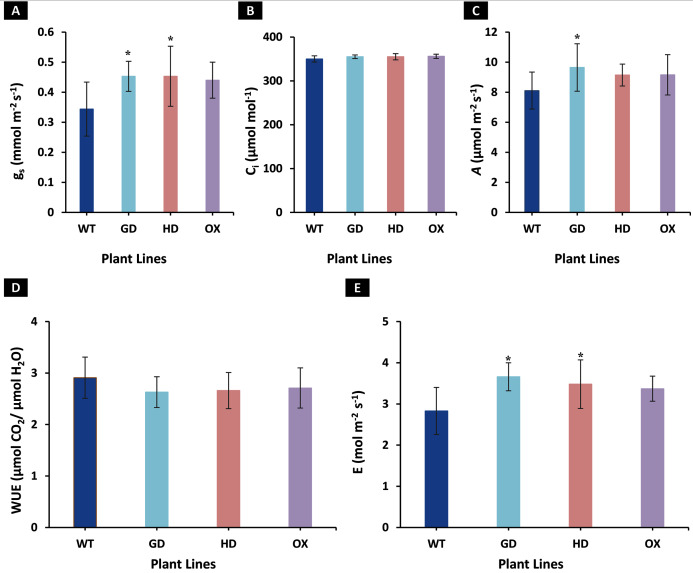
Effect of cyanobacterial glycolate decarboxylation pathway genes on gas exchange parameters in transgenic *Arabidopsis thaliana* plants. Transgenic plants were grown in soil and after 8 weeks were utilized for gas exchange measurements by using LI-6400 portable photosynthesis system. (A–E) Evaluating photosynthesis related parameters in transgenic plants. Different gas exchange parameters including stomatal conductance (g_s_), intercellular CO_2_ (C_i_), net photosynthetic rate (*A*), water use efficiency (WUE), and transpiration rate (E) were measured in 8-week-old soil-grown GD, HD, and OX plants and compared with wild type (WT) plants. *t test* was performed to evaluate the significant difference among measured traits in the transgenic lines, compared to WT plants. Data has been presented as mean ± standard deviation of six biological replicates originating from same experiment, where asterisk (*) denotes the significant difference at *P < 0.05*. (A) Effect on g_s_ in GD, HD, and OX lines as compared to WT, (B) Effect on C_i_ in GD, HD, and OX lines as compared to WT, (C) Effect on *A* in GD, HD, and OX lines as compared to WT, (D) Effect on WUE in GD, HD, and OX lines as compared to WT, and (E) Effect on E in GD, HD, and OX lines as compared to WT.

To identify the effect of transformations with individual bypass genes on photosynthesis, gas exchange measurements were made on 8-week-old soil-grown plants. Different photosynthetic and related parameters including stomatal conductance (g_s_), intercellular CO_2_ (C_i_), net photosynthetic rate (*A)*, water use efficiency (WUE), and transpiration rate (E) were measured in eight-week-old transgenic (GD, HD, and OX) and wild type (WT) plants. An increase of 24% in GD (*P* = 0.011), 23% in HD (*P* = 0.037), and 21% in OX (*P* = 0.084) plants in g_s_ was observed, respectively as compared to WT plants (Fig. 2A). Despite increase in g_s_, no change in C_i_ was observed among transgenic plants (GD, HD, and OX) as compared to WT plants (Fig. 2B). Among transgenic plants, GD, HD, and OX exhibited 16% (*P* = 0.035), 11% (*P* = 0.066), and 11.5% (*P* = 0.17) increase in *A*, respectively as compared to WT plants (Fig. 2C). A non-significant decrease was observed for WUE among transgenic plants (GD: −9%, HD: −8%, OX: −7%) as compared to WT plants (Fig. 2D). However, an enhancement of 22.7% in GD (*P* = 0.002), 19% in HD (*P* = 0.026), and 16.3% in OX (*p* = 0.075) plants was observed in E in comparison to WT plants (Fig. 2E). Our results indicated that independent expression of cyanobacterial glycolate decarboxylation pathway genes improved g_s_ and an associated enhancement of *A*.

### Transgenic plants independently expressing GDH, HDH, and ODC exhibited improved light reaction efficiency

**Figure 3:**
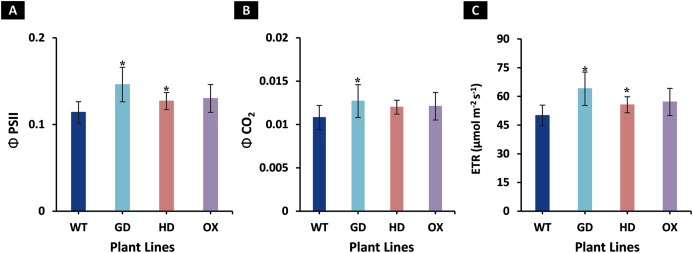
Quantum efficiencies and chlorophyl fluorescence-based electron transport rates. Different light and gas exchange parameters were measured in the transgenic plants by using leaf chamber fluorimeter. (A–C) Analysis of fluorescence related parameters in *Arabidopsis thaliana* transgenic plants. The soil grown transgenic plants were utilized to determine light adapted quantum efficiency of photosystem II (ΦPSII), quantum efficiency of CO_2_ assimilation (ΦCO_2_), and chlorophyl fluorescence-based photosynthetic electron transport rate (ETR). *T test* was performed to evaluate the significant difference among transgenic lines for the measured traits, compared to WT plants. Data has been presented as mean ± standard deviation of six biological replicates originating from same experiment, where asterisk (*) denotes the significant difference at *P < 0.05*. (A) Effect of single cyanobacterial gene on ΦPSII in GD, HD, and OX lines as compared to WT, (B) Effect of single cyanobacterial gene on ΦCO_2_ in GD, HD, and OX lines as compared to WT, and (C) Effect of single cyanobacterial gene on ETR in GD, HD, and OX lines as compared to WT.

**Figure 4:**
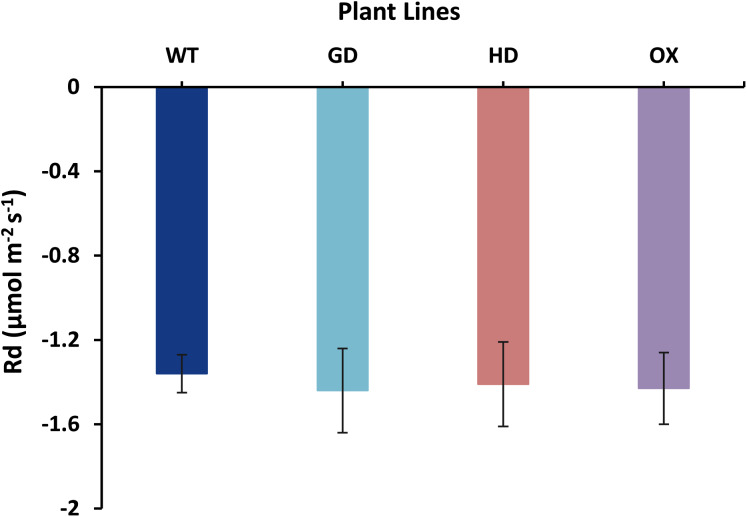
Effect of cyanobacterial genes on respiration rate (Rd) in the dark. Respiration rate was measured in transgenic lines as well as WT plants by using leaf chamber fluorimeter. Comparison of dark respiration rates between the different genotypes used in the study. Data has been presented as mean ± standard deviation of six biological replicates originating from same experiment.

The increased photosynthesis in transgenic lines led us to evaluate the influence of independently expressed cyanobacterial GDH, HDH, and ODC genes on fluorescence related parameters using 8-week-old soil-grown plants to assess any impacts on the operation of PS2. Different light and gas exchange related parameters including light adapted quantum efficiency of photosystem II (ΦPSII), photosynthetic electron transport rate (ETR), quantum efficiency of CO_2_ assimilation (ΦCO_2_), and respiration rate (Rd) in the dark were measured. Among the transgenic plants examined, GD showed the highest increase in ΦPSII followed by OX and HD (Fig. 3A). A 22% (*P* = 0.001), 12% (*P* = 0.020), and 10% (*P* = 0.084) increase was observed in GD, OX, and HD plants, respectively, in comparison to WT plants (Fig. 3A). For ΦCO_2,_ 15% (*P* = 0.019), 10.5% (*P* = 0.071), and 10.5% (*P* = 0.162) increase was observed in GD, HD, and OX transgenic plants, respectively, as compared to WT plants (Fig. 3B). Furthermore, the installation of the cyanobacterial genes also led to the enhancement in ETR such that GD, HD and OX plants exhibited a 21.5% (*P* = 0.001), 10% (*P* = 0.021), and 12.4% (*P* = 0.086) increase as compared to WT plants (Fig. 3C). In case of Rd, a non-significant increase was observed at *P < 0.05* such that in comparison to WT plants GD, HD, and OX plants showed 5%, 3.5%, and 5% increase in Rd (Fig. 4). These results suggest that the cyanobacterial genes can improve the quantum efficiency in transgenic plants.

### Single gene transformation altered the relative photorespiration rate in transgenic plants

**Figure 5:**
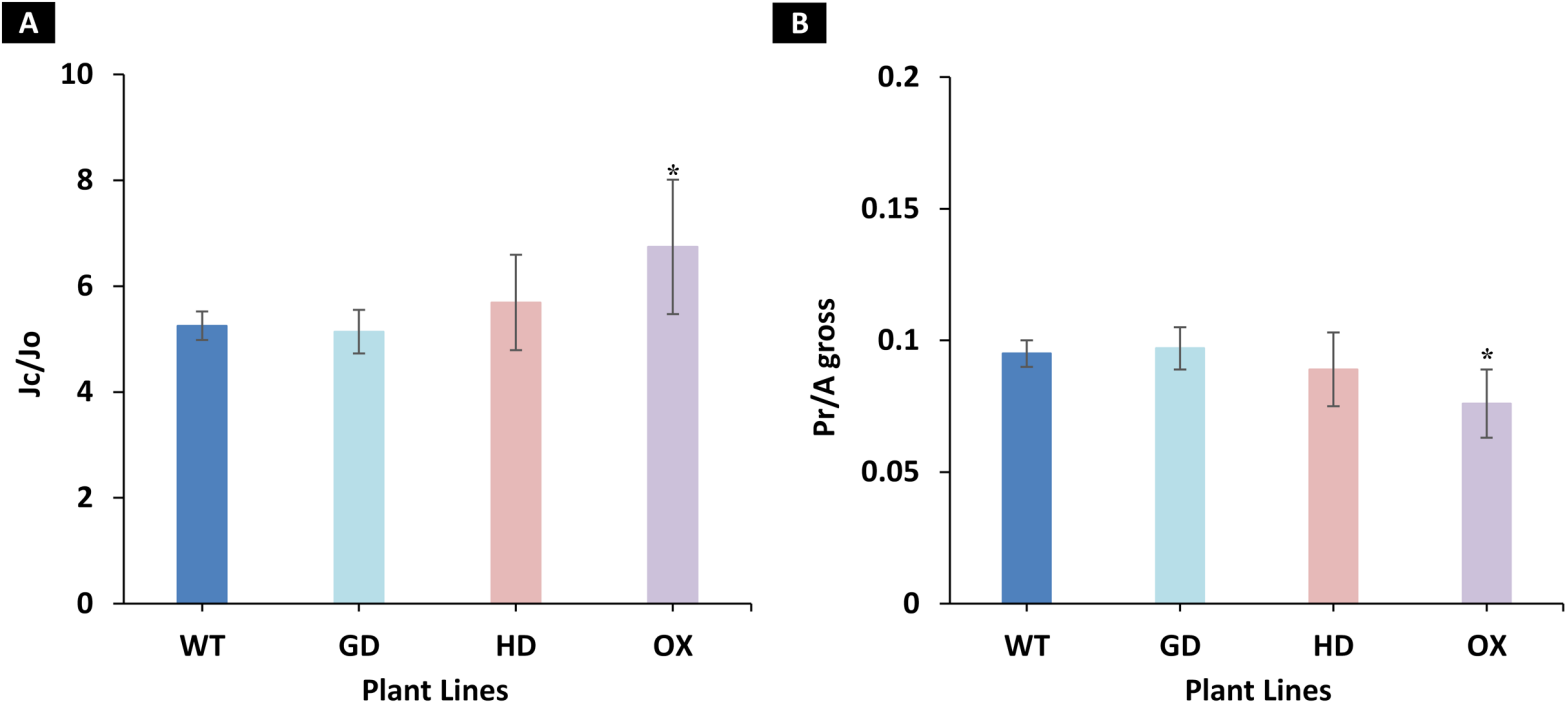
Ratio of electron flows attributable to carboxylative and oxygenative reactions and rate of photorespiration relative to gross photosynthesis. (A), Comparison of the ratio of the electron flow rate devoted to RuBP carboxylation (J_C_) over the electron flow rate devoted to RuBP oxygenation (J_O_) between the different genotypes used in the study. (B), Comparison of the rates of photorespiration (Pr) relative to gross photosynthesis (A_gross_). Data has been presented as mean ± standard deviation of six biological replicates originating from same experiment, where asterisk (*) denotes the significant difference at *P < 0.05*.

Among the transgenic plants, GD plants showed the rate of photorespiration relative to gross photosynthesis (Pr/A_gross_) and the ratio to electron rates attributable to carboxylation and oxygenation reactions (J_C_/J_O_) comparable to WT plants (Fig. 5). Similarly, HD plants showed small, yet non-significant decrease (*P* = 0.19) in Pr/A_gross_ and increase in J_C_/J_O_ (*P* = 0.14) as compared to WT plants (Fig. 5). In contrast, the OX plants displayed significant decrease (*P* = 0.004) in Pr/A_gross_ and increase in J_C_/J_O_ (*P* = 0.01) relative to the WT plants, which indicates a possible partial photorespiratory bypass resulting from the single gene transformation (Fig. 5). These results suggest that the single gene has differently affected photorespiration in the transgenic plants.

### Transgenic plants showed improved biomass accumulation

**Figure 6:**
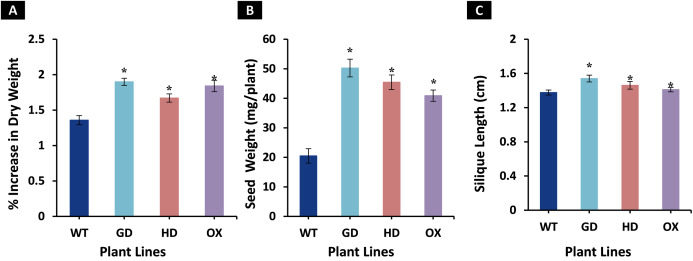
Growth analysis of transgenic lines expressing cyanobacterial GDH, HDH and ODC. Plants were grown in soil and harvested at mature stage to determine growth parameters. (A–C) Analysis of biomass accumulation in transgenic lines. Aboveground biomass of mature plants was harvested and then dried to determine phenotype and seed dry weight. *t test* was performed to evaluate the significant difference in biomass accumulation among transgenic lines, compared to WT plants. Data has been presented as mean ± standard deviation of six biological replicates originating from same experiment, where asterisk (*) denotes the significant difference at *P < 0.05*. (A) % increase in plant dry weight in transgenic lines in comparison with WT, (B) Enhancement in seed dry weight in transgenic lines in comparison with WT, and (C) Increase in silique length in transgenic lines in comparison with WT.

The enhancement observed in photosynthetic and fluorescence parameters prompted us to evaluate the effect of GDH, HDH and ODC genes on seed and plant biomass accumulation. Total biomass of mature plants was harvested and dried for further analysis. All transgenic plants exhibited a significant increase in dry weight as compared to WT plants (Fig. 6A). Among transgenic plants, a 28.5 % (GD), 18.7% (HD), and 26% (OX) increase was observed as compared to WT plants (Fig. 6A). Next, the effect of foreign genes on seed quantity was also determined using seeds from dried siliques (Fig. 6B). In comparison to WT plants, GD, HD, and OX plants exhibited a significant increase of 59%, 55%, and 50% in seed weight, respectively (Fig. 6B). Silique length was also measured, and all the transgenic plants showed a significant increase, GD plants showed a 11% increase followed by HD (6.2%), and OX (2.6%) plants (Fig. 6C). Taken together, these results suggest that the introduction of these genes into C_3_ plants can enhance plant biomass.

### Introduction of cyanobacterial genes improved the sugars and starch contents in plants

**Figure 7:**
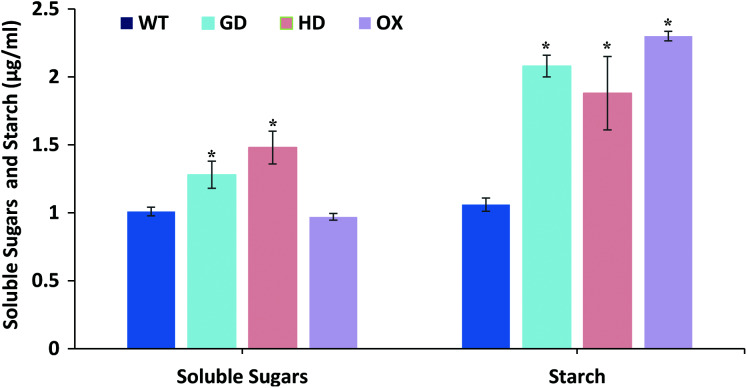
Quantification of soluble sugars and starch content in transgenic *Arabidopsis thaliana* plants. The soluble sugars and starch content was quantified by using leaf extracts of transgenic and WT plants. Data has been presented as mean ± standard deviation of six biological replicates, where asterisk (*) denotes the significant difference at *P < 0.05*.

The overall increase in biomass accumulation led us to evaluate the improvement in sugars and starch content. Plants samples were dried, and extracts were made to quantify the sugars and starch. The soluble sugars were significantly increased *i.e*. 21% (GD plants) and 31% (HD plants), while OX plants showed a 4% decrease in comparison to WT plants (Fig. 7). In the case of starch content, there were significant, 49% (GD plants), 33% (HD plants), and 54% (OX plants) increases compared to WT (Fig. 7). Our results suggest that the improvement in photosynthesis has increased the sugars and starch content which positively influenced the biomass accumulation.

### Transgenic plants exhibited improved stress tolerance response

**Figure 8:**
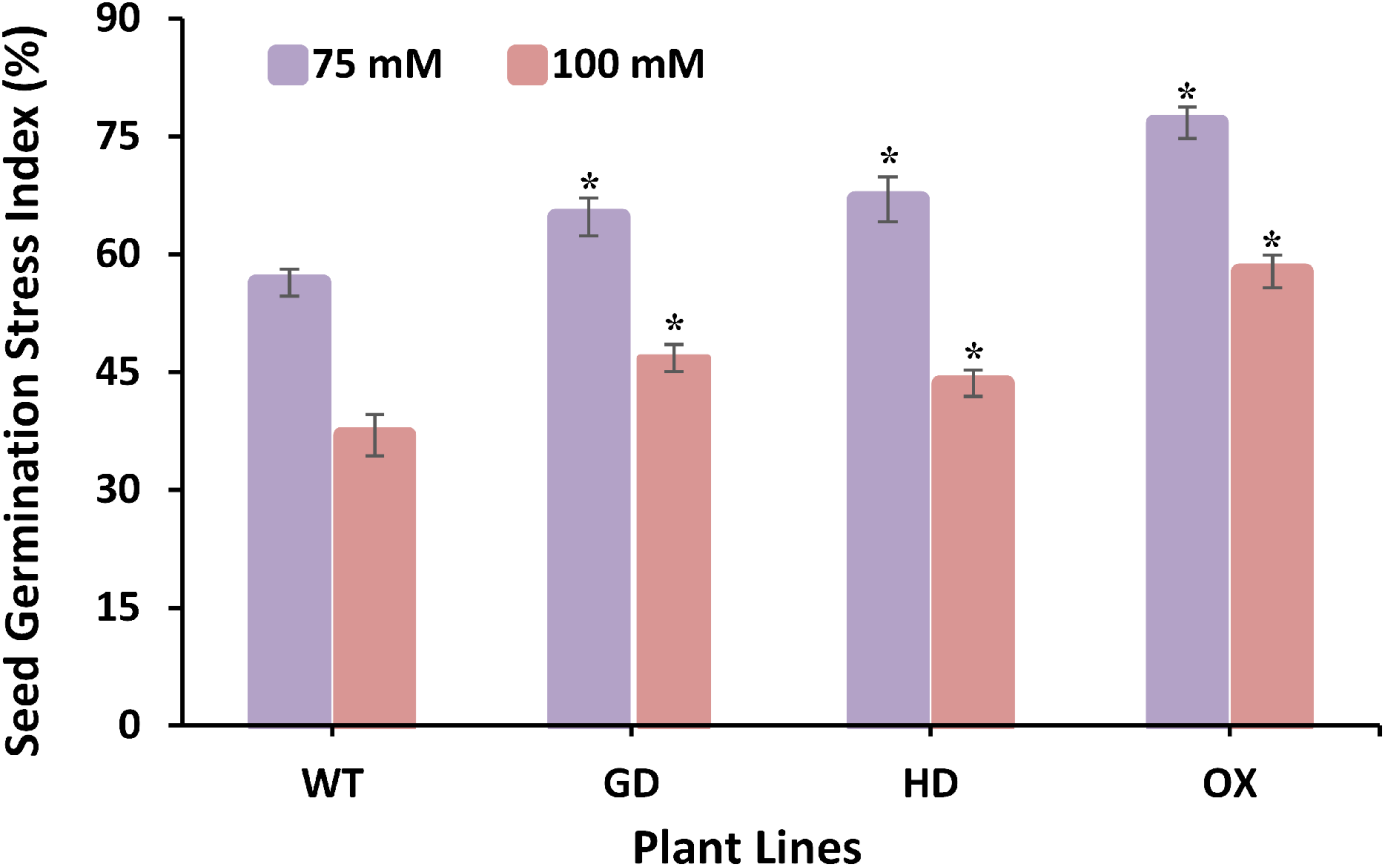
Effect of salt stress on seed germination of transgenic lines expressing cyanobacterial genes independently. Transgenic lines as well as WT plants were grown in media supplemented with two different concentrations of sodium chloride (NaCl) *i.e*. 75 mM and 100 mM. Seed germination stress index (SGSI) was then measured in treated transgenic and WT plants. *t test* was performed to evaluate the significant difference in SGSI among transgenic lines, compared to WT plants. Data has been presented as mean ± standard deviation of six biological replicates, where asterisk (*) denotes the significant difference at *P < 0.05*. SGSI of WT, GD and HD lines as evaluated against different levels of salt stress.

The improvement in photosynthesis and plant biomass accumulation due to the independent expression of GDH and HDH genes under ambient conditions prompted us to evaluate their response under stress conditions by exposing them at different salinities. Growing them on 75 mM NaCl media, GD, HD, and OX exhibited a significant 13%, 15.9%, and 25.5% increase in the germination index (GI) as compared to WT plants (Fig. 8). At a higher salt concentration *i.e*. 100 mM, there was a significant 20%, 15%, and 36% increase in Gi was observed in GD, HD, and OX plants, respectively (Fig. 8). Our results suggest that the foreign genes have not only enhanced the photosynthetic efficiency but also improve the stress tolerance level in plants.

### Principal component analysis revealed influence of photosynthetic and fluorescence parameters towards enhancing biomass accumulation

**Figure 9:**
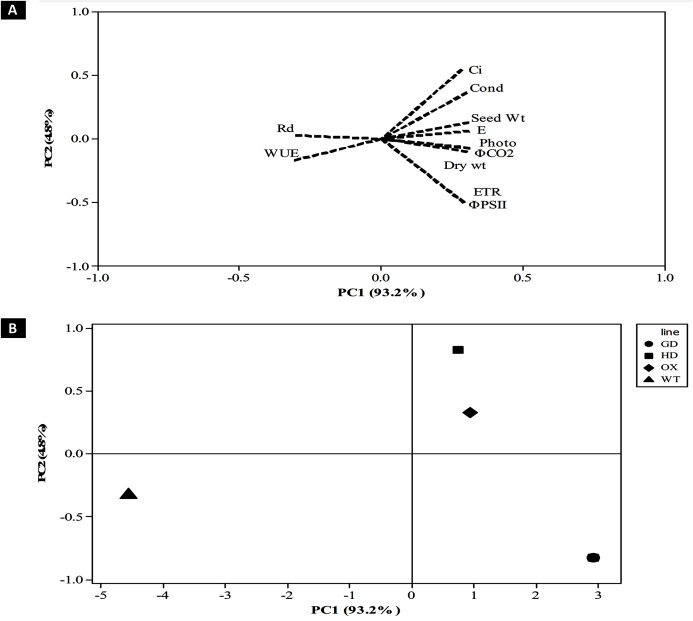
Principal component analysis (PCA) of transgenic lines. PCA was performed using minitab software to evaluate the influence of photosynthetic, fluorescence and growth-related parameters in overall variation observed in transgenic lines. (A–B) Contribution of traits in generating overall variation. Analysis was performed by standardizing all traits and then studied at PC1 and PC2. (A) Eigenvalues of traits in PC1 and PC2 and (B) categorization of plants based on contribution of traits in overall variation.

After evaluating the photosynthetic and fluorescence parameters independently, we set out to elucidate the relative influence of these parameters on the enhancement of photosynthesis and biomass accumulation. For that, principal component analysis (PCA) was performed to analyze the weightage of each parameter (Fig. 9). A total of 98% of the variation was found for the first two principal components *i.e*. PC1 and PC2. Among the different photosynthetic and fluorescence traits studied, the most significant for the PC1 were *A*, g_s_, ΦPSII, ΦCO_2_, ETR, and E. While C_i_ was the significant positive contributor for the PC2. Finally, the WUE and Rd was found to be least significant contributor for both the PC1 and PC2. Furthermore, the contribution of different parameters towards enhancing photosynthesis also led to the categorization of transgenic plants indicating the influence of each cyanobacterial gene (Fig. 9B). Among transgenic lines, GD plants ranked higher followed by OX, and then HD in comparison to WT (Fig. 9B). All the variables measured and their contribution to the PCA are given in Table 1.

**Table 1:** Principal component analysis of plant growth, photosynthetic and fluorescence related parameters.

Variables	PC1	PC2
Net Photosynthesis Rate (*A*)	0.312	−0.071
Stomatal Conductance (g_s_)	0.3	0.363
Water Use Efficiency (WUE)	−0.306	−0.169
Intercellular CO_2_ (C_i_)	0.283	0.545
*φ*PSII	0.291	−0.502
Dark Respiration (Rd)	−0.303	0.027
*φ*CO_2_	0.312	−0.075
Electron Transport Rate (J_max_)	0.291	−0.498
Transpiration Rate (E)	0.309	0.062
Dry weight	0.301	−0.098
Seed Weight	0.308	0.131

### Transcriptome analysis of GD plants showed differentially expressed genes in different plant metabolic pathways

**Figure 10:**
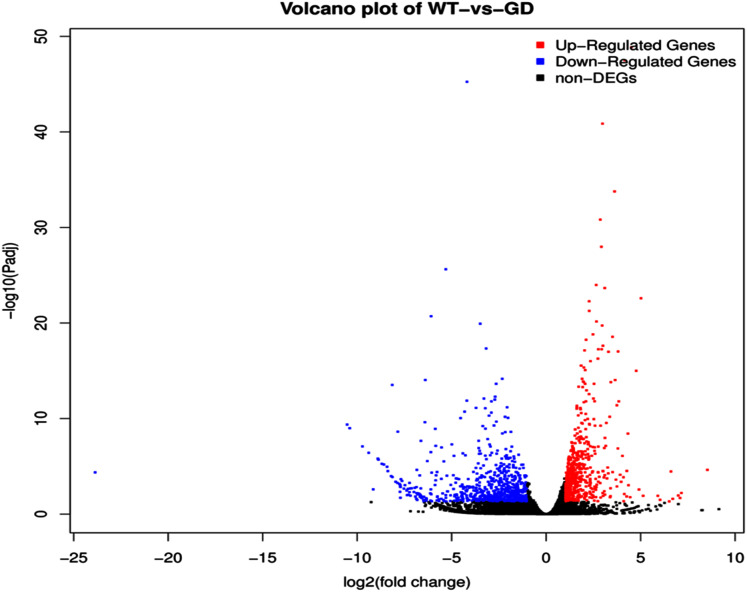
Differentially expressed genes (DEG) in response to integration of glycolate dehydrogenase (GDH) in *Arabidopsis thaliana*. Transcriptomic analysis was performed to identify DEGs in the transgenic plants. Expression profile of different metabolic pathway genes in response to integration of cyanobacterial gene. Volcano map was generated to reveal up and down regulated genes in the GD lines.

**Figure 11:**
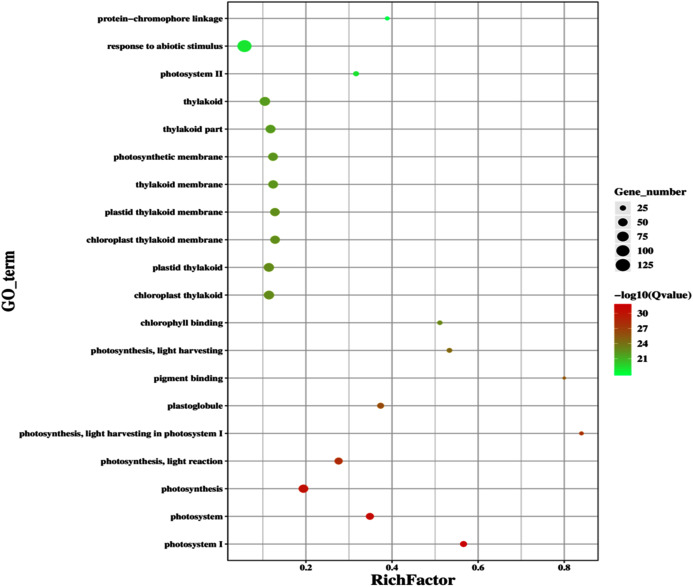
Classification of upregulated genes in the GD plants. Gene classification into different pathways were done by using annotation available at KEGG. GO enrichment classification of upregulated genes. The rich factor denotes the ratio of differentially expressed genes in a pathway term to the total genes annotated in the same pathway term. The size of the dot depicts the number of genes enriched in a particular pathway term. The q-value is a corrected *p*-value, the lower q-value represents higher degree of pathway enrichment.

**Figure 12:**
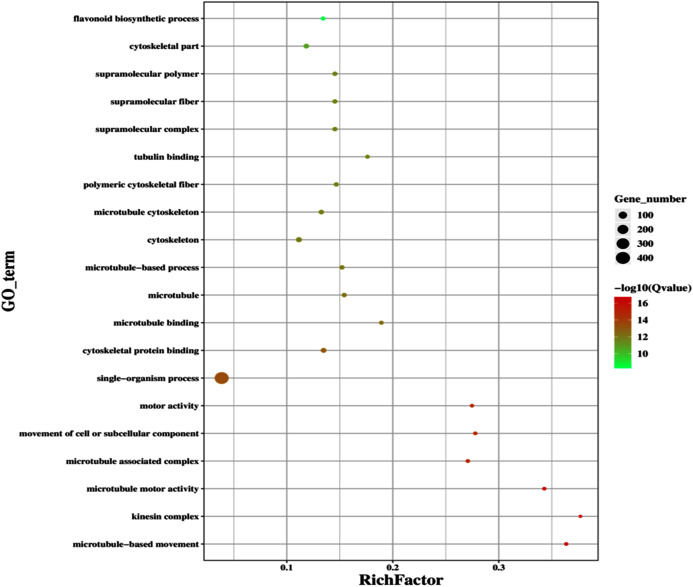
Classification of downregulated genes in the GD plants. Gene classification into different pathways were done by using annotations available at KEGG. GO enrichment classification of downregulated genes. The rich factor denotes the ratio of differentially expressed genes in a pathway term to the total genes annotated in the same pathway term. The size of the dot depicts the number of genes enriched in a particular pathway term. The q-value is a corrected *p*-value, the lower q-value represents higher degree of pathway enrichment.

**Figure 13:**
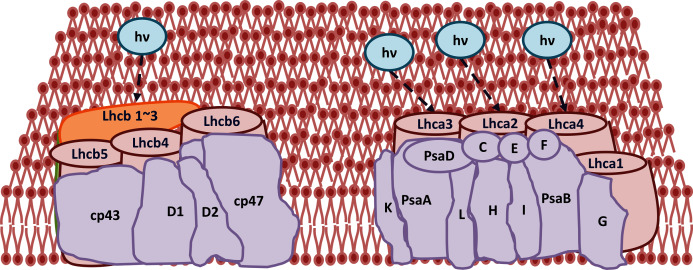
Transcriptomic profiling of light harvesting complex (Lhc) genes in GD transgenic plants. The transcriptomic analysis revealed an upregulation in Lhc, a, and b genes due to the integration of cyanobacterial glycolate dehydrogenase (GDH) gene.

The improvement in photosynthetic parameters in GD plants prompted us to evaluate the effect of GDH on other genes which have contributed towards enhancement in biomass accumulation. Transcriptomic profiling of GD plants was carried out and the differentially expressed genes (DEG) were evaluated based on the calculation of FPKM (fragment per kilobases per million). The transcriptomic data was validated through real time RT-PCR of selected genes (data not shown). In total, 1,425 genes were differentially expressed in GD plants, out of which 650 genes were up regulated while 775 genes were down regulated as shown in the volcano map (Fig. 10). The DEGs were categorized into twenty gene ontology (GO) terms for both up regulated and down regulated genes (Figs. 11, 12). Maximum number of genes were over expressed related to abiotic stresses while the overexpression level was high in genes related to photosynthesis and photosystems (Fig. 11). In case of down regulation, maximum number of genes related to single-organism processes were downregulated, however, the highest down regulation in expression was observed in cytoskeleton and microtubules (Fig. 12). The KEGG analysis exhibited upregulation of light harvesting complex (Lhc, a, and b) from photosystem 1 and II (Fig. 13). The genes related to the light harvesting chlorophyll protein complex a (Lhca) were AT3G54890 (Lhca1), AT3G61470 (Lhca2), AT1G61520 (Lhca3), and AT3G47470 (Lhca4) while the genes related to Lhcb were AT2G34430 (Lhcb1), AT2G05100 (Lhcb2), AT5G54270 (Lhcb3), AT3G08940 (Lhcb4), AT4G10340 (Lhcb5), and AT1G05207 (Lhcb6), respectively (Fig. 13). The transcriptomic profiling of GD plants also revealed that genes related to glyoxylate and related metabolic processes, chlorophyll biosynthesis, sugar and starch metabolism, and plant antioxidative defense mechanism were also upregulated (Table 2). Taken together, these results indicate that the engineering of GDH has also affected the plant transcriptome, which improved plant growth and stress tolerance.

**Table 2:** Description of important up regulated genes in GD plants and their functions.

Gene ID	Name	Function	Log Fold Change	Reference (related to function)
AT5G13630	Magnesium chelatase	Such enzymes are involved in Mg^+2^ insertion in protoporphyrin during chlorophyll biosynthesis.	1.32	Mochizuki et al., 2001
AT3G56940	Putative dicarboxylate diiron	Involved in chlorophyll biosynthesis.	1.4	Kauss et al., 2012
AT1G44446	Chlorophyllide a oxygenase	This enzyme is also involved in chlorophyll biosynthesis and performs committed step of conversion of chlorophyllide a to chlorophyllide b. Lack of function mutants are deficient in chlorophyll b, thus hampering light harvesting by photosystem II.	1.62	Yang et al., 2016
AT1G74470	Geranylgeranyl reductase activity	It is also involved in chlorophyll biosynthesis, which converts geranylgeranyl-chlorophyll to chlorophyll a.	1.21	Lange & Ghassemian, 2003
AT3G13790	Cell wall invertase/Glycosyl hydrolase	These proteins are involved in carbon partitioning and cleave sucrose into glucose.	1.47	Minic, 2008
AT3G60130	Beta glucosidase 16	It is involved to provide intermediates during cell wall development such as lignification.	1.1	Sing, Verma & Kumar, 2016
AT2G18700	Trehalose phosphatase/synthase	This gene encodes a protein which is putatively involved in trehalose biosynthesis, which is also involved in osmotic regulation during stress.	1.93	Ponnu, Wahl & Schmid, 2011
AT1G60140	Trehalose phosphate synthase	This gene encodes a protein which is putatively involved in trehalose biosynthesis, which is also involved in osmotic regulation during stress	1.44	Ponnu, Wahl & Schmid, 2011
AT1G23870	Trehalose -6-phosphatase synthase	This gene encodes a protein which is putatively involved in trehalose biosynthesis, which is also involved in osmotic regulation during stress	1.41	Ponnu, Wahl & Schmid, 2011
AT5G55700	Beta amylase 4	This gene encodes a protein which is involved in starch breakdown and localized in chloroplasts.	1.1	Thalmann et al., 2019
AT2G13360	Alanine: glyoxylate aminotransferase	Involved in photorespiration that performs transamination of glyoxylate.	1.03	Liepman et al., 2019
AT1G23310	Glyoxylate aminotransferase	It is involved in glycine biosynthesis and may have localization in peroxisomes and chloroplasts.	1.4	Liepman et al., 2019
AT1G68010	Hydroxy pyruvate reductase	It is involved in oxidative photosynthetic pathway, specifically synthesis of glycerate biosynthesis.	1.03	Timm et al., 2011
AT1G55920	Serine acetyltransferase	Involved in cysteine biosynthesis in chloroplasts and cytosol.	1.1	Tavares et al., 2015
AT5G50950	Fumarase	It is reportedly located in chloroplasts, cytosol and mitochondria. It is involved in fumarate synthesis from malate in leaves and help to promote nitrogen assimilation.	1.4	Nunes-Nesi et al., 2007
AT5G18170	Glutamate dehydrogenase 1	It has mitochondrial localization and supposedly involved in ammonia assimilation.	1.54	Labboun et al., 2009
AT1G13700	6-Phosphogluconolactonase-1	Involved in pentose phosphate pathway. Important source of NADPH-a reducing equivalent.	1.52	Xiong et al., 2009
AT4G39640	Gamma-glutamyl trans peptidase 1	It is present in leaves and degrades glutathione. It is also involved to relieve oxidative stress by metabolizing GSH-glutathione.	1.2	Gong et al., 2005
AT2G02930	Glutathione S-Transferase 16	Member of ubiquitous large gene family involved in mitigation of wide range of stresses predominately oxidative stress.	1.35	Gong et al., 2005
AT1G10360	Glutathione S-Transferase 29	Member of ubiquitous large gene family involved in mitigation of wide range of stresses predominately oxidative stress.	1.1	Gong et al., 2005
AT2G25080	Glutathione peroxidase 1	It is involved in alleviation of oxidative stress through peroxidase activity.	1.84	Hasanuzzaman et al., 2017
AT4G35090	Peroxisomal catalase	Also named as cat2-1, which is involved in H_2_O_2_ detoxification in peroxisomes. The loss of function mutant showed elevated H_2_O_2_ levels.	1.91	Mhamdi et al., 2010

## Discussion

Plants being the natural carbon sequesters, employ photosynthesis to capture atmospheric CO_2_ and convert it into aboveground and belowground biomass (Ruan, Shao & Teixeira Da Silva, 2012). It is well known that the process of photosynthesis can be compromised by photorespiratory activity in C_3_ plants, which is exacerbated under stress conditions (Dusenge, Duarte & Way, 2019).

In addition to installing a complete photorespiratory bypass, transformations involving a single gene has positively contributed to an enhancement of plant biomass in different C_3_ plants including *Solanum tuberosum, Camelina sativa* and *Arabidopsis thaliana* (Kebeish et al., 2007; Nölke et al., 2014; Dalal et al., 2015; Ahmad et al., 2016; Cui et al., 2016; Hagemann & Bauwe, 2016). Although the single gene transformation resulted in an improvement in plant biomass the underlying mechanism(s) contributing to this enhancement is largely unknown. Therefore, the role of single gene-associated photorespiratory bypasses in the enhancement of plant growth needs to be elucidated. We recently reported *Arabidopsis thaliana* independently expressing cyanobacterial glycolate decarboxylation pathway (Fig. 1) genes *i.e*. glycolate dehydrogenase (GDH: GD plants), hydroxyacid dehydrogenase (HDH: HD plants), and oxalate decarboxylase (ODC: OX plants) (Bilal et al., 2019). The transgenic lines exhibited the expression of the introduced transgenes, increasing growth, and chlorophyll concentration. These results led us to further evaluate these lines for different photosynthetic, fluorescence and growth-related responses, as well as investigating their transcriptomic profile (GD plants), in order to understand the effect of a single gene on the plant transcriptome.

The transgenic plants exhibited improved photosynthetic parameters, including a higher stomatal conductance (g_s_), net photosynthesis (*A)*, and transpiration rate (E). However, the intercellular CO_2_ (C_i_) concentration and water use efficiency (WUE) of transgenic plants was comparable to wild type (WT) plants (Fig. 2). Transgenic plants also showed improvement in light adapted photosystem II (ΦPSII) quantum efficiency, quantum yield of CO_2_ assimilation (ΦCO_2_), photosynthetic electron transport rate (ETR), and dark respiration rate (Rd) as compared to WT (Figs. 3, 4). Importantly, the OX plants also displayed a reduced relative rate of photorespiration (Fig. 5B) compared to WT plants which hints at a functional, yet partial, photorespiratory bypass as a result of single gene (ODC) transformation. Furthermore, the transformed genes led to a higher biomass accumulation, increased seed production, and silique length in transgenic plants as compared to the WT (Fig. 6). Additionally, the soluble sugar and starch content were also higher in the transgenic lines (Fig. 7). Interestingly, transgenic plants also exhibited a higher seed germination stress index under salt stress conditions (Fig. 8). Additionally, principal component analysis (PCA) revealed the contribution of the studied parameters, including g_s_, C_i_, *A*, E, ΦPSII, ΦCO_2,_ ETR, and WUE towards the overall variation observed in photosynthesis and biomass accumulation (Fig. 9). Furthermore, the transcriptomic profiling showed differentially expressed genes that were both up and down regulated in GD plants (Figs. 10–13).

Our study revealed that the GD plants exhibited a significant increase in *A* as well as g_s_, while a non-significant increase in *A* and g_s_ was observed in HD, and OX plants only showed a significant increase in g_s_ (Fig. 2). An increase in *A* can be attributed to the enhanced sequestration of atmospheric CO_2_ (C_a_) due to a higher g_s_ (Fig. 2). A positive correlation exists between g_s_ and *A* such that a higher g_s_, will be associated with a higher *A* (Faralli, Matthews & Lawson, 2019). Furthermore, stomatal aperture is under the control of external and internal ques which can modulate CO_2_ uptake and downstream diffusion towards the chloroplast (Faralli, Matthews & Lawson, 2019). In the transgenic plants, there is a need to further elucidate the contribution of the components of stomatal aperture including pore size and internal cues in causing an increase in g_s_ which were associated with a higher *A*. In addition to this, there is a need to examine the nutrient requirements of transgenic plants expressing partial or complete bypasses under ambient atmospheric CO_2_ (C_a_) levels as modifications in N availability or allocation could lead to an enhancement of *A*. In a kinetic bypass model study, we evaluated the effect of the complete glycolate decarboxylation pathway on *A* and reported increase in *A* at ambient C_i_ concentrations, but in the *A*-Ci curve, the bypass model attained steady state even at 380 ppm and further increases in C_i_ up to 950 ppm had no effect on *A* (Khurshid et al., 2020). Furthermore, we identified that the higher C_i_ requires more inorganic phosphate (Pi) to cause any further increase in *A*, which was shown in both the C_3_ and bypass models (Khurshid et al., 2020).

Previous studies reported the catabolization of glyoxylate (GOA) through enzymes such as pyruvate dehydrogenase complex (PDC) within the chloroplast resulted in CO_2_ release within the chloroplast (Kearney & Tolbert, 1962; Kisaki & Tolbert, 1969; Blume et al., 2013). Oliver & Zelitch (1977) reported enhancement in photosynthesis in the GOA treated tobacco leaf discs mainly because of enhanced CO_2_ fixation and reduced GCA level, which is indicative of lower oxygenative photosynthesis (Oliver & Zelitch, 1977). In a latter study, the soyabean mesophyll cells treated with GOA has also been reported that lower concentrations of CO_2_ were required for the half maximal velocity of Rubisco which resulted in improved photosynthesis, thereby decreasing the oxygenative reaction and affected GCA synthesis (Oliver, 1980). In line with these reports, the GDH expressing transgenic potato plants exhibited enhanced GOA contents through breakdown of GCA in the chloroplast (Ahmad et al., 2016). The same gene (GDH) was introduced into *Arabidopsis thaliana* which could have produced the similar results in GD plants. Dalal et al. (2015) has also reported an increase in GOA content and photosynthesis in GDH-expressing *Camelina Sativa* plants. Interestingly, HD plants also showed an increase in *A* (Fig. 2C), which likely suggests the catabolization of GCA by HDH. Though the enzymatic activity of HDH in transgenic *Arabidopsis* plants was not measured, 2-hydroxyacid dehydrogenases (2-HDH), are ubiquitous enzymes having the ability to catabolize multiple substrates in humans as well as in plants (Baker et al., 2004). The enzyme HDH has been implicated in the catabolism of GCA into GOA, which is then converted into oxalate in humans (Baker et al., 2004). Eisenhut et al. (2008) has also reported the catabolism of GOA by HDH in the cyanobacterial glycolate decarboxylation pathway. Considering the role of HDH reported in the literature, we speculate that HDH has utilized GCA as a substrate and catabolized it, causing an increase in *A* (Fig. 2C), but this needs to be further evaluated. The other enzyme, ODC, is involved in the cyanobacterial decarboxylation pathway, where it catabolizes oxalate into formate and releases one molecule of CO_2_ as well (Eisenhut et al., 2008). Despite the enhancement observed in OX transgenic lines (Fig. 2C), the role of ODC in GCA catabolism and biomass accumulation needs further investigation to identify the role of ODC in plant metabolism and photosynthesis.

Photosystem II (PSII) is a highly conserved protein complex, which drives the light reaction of photosynthesis by extracting electrons from water (Liu et al., 2019). It is well documented that a linear relationship exists between ΦPSII and ΦCO_2_ (Cheng, Fuchigami & Breen, 2001). ETR depends upon PSII quantum yield and has also been reported as a direct determinant of *A* (Kubota & Yoshimura, 2002). Our results indicate that the increases in *A*, in GD (significant) and HD and OX plants (non-significant), are associated with an enhancement in ΦPSII, ΦCO_2_ and ETR (Fig. 3) (Cheng, Fuchigami & Breen, 2001; Kubota & Yoshimura, 2002).

It has also been reported that bypass genes led to quick floral induction and plants produced more siliques, which resulted in more seeds being produced (Dalal et al., 2015). Transcriptomic analysis also revealed an enhanced expression of sugar transporters which would increase the transport of photosynthate produced during photosynthesis to the sink cells, thereby increasing seed yield (Dalal et al., 2015). In agreement with this report, our transgenic plants also exhibited a significant increase in silique length in GD and HD plants, although the effect was not—significant in OX plants, and this has contributed to a higher seed weight (Fig. 6).The increase in seed weight can also be correlated to an increase in cauline branches of transgenic *Arabidopsis thaliana* plants expressing cyanobacterial genes, as reported in our previous study (Bilal et al., 2019). Additionally, we also observed an enhancement in seed size based upon the 1,000 seed weight (data not shown). However, further work would need to be carried out on the underlying reason(s) for the increase in seed size and stored contents. The previous studies on photorespiratory complete as well as partial bypass reported an interesting phenomena that biomass accumulation enhanced almost at double rate than *A* (Kebeish et al., 2007; Dalal et al., 2015). It needs to be noted that the increased biomass/seed production in our transgenic plants clearly indicates an increased capacity for CO_2_ fixation, as photosynthesis provides all the carbon necessary for plant growth. This increased capacity appears to be the result of either (a) photosynthetic upregulation with no change in the relative rate of photorespiration (*i.e*. GD plants), (b) the result of a decrease in the relative rate of photorespiration even without a significant photosynthetic upregulation (*i.e*. OX plants) or (c) a combination of a small photosynthetic upregulation and a small decrease in the relative rate of photorespiration (*i.e*. HD plants).

Engineering of photorespiratory bypasses, complete as well as partial, and modifications in other genes, such as phosphophenol pyruvate carboxylase (PEPC), cytochrome c_6_ (Cyt c_6_), and pyruvate phosphate dikinase (PPDK) have resulted in improved photosynthesis, as well as an enhancement in soluble sugars and starch (Kebeish et al., 2007; Xia & Cao, 2013; Nölke et al., 2014; Dalal et al., 2015; Yadav et al., 2018; Yadav, Rathore & Mishra, 2020). Nölke et al. (2014) have also reported an enhancement in sugar contents by transforming GDH alone in potato. Furthermore, Dalal et al. (2015) has also related the enhancement in *A* with an increased level of starch production. In our case, soluble sugars were significantly increased in the GD and HD transgenic plants while starch content was significantly enhanced in all the transgenic plants (Fig. 7). Gago et al. (2016) has also reported that sugars and organic acids can influence g_s_ and positively affect *A* (Gago et al., 2016). Interestingly, OX showed a decline in sugars, although there was an increase in starch content and the reason for this needs further investigation.

Seed germination is a crucial stage which is required for successful growth of plants (Eastmond & Graham, 2001). Seeds need to be able to utilize stored reserves and convert them into a utilizable form to support plant growth (Bewley & Black, 2013). The glyoxylate cycle has also been reported in seeds and is utilized to generate energy in the form of ATP (Eastmond & Graham, 2001). Stressful environmental conditions cause deleterious effects on seed germination (Duan et al., 2014). Cui et al. (2016) overexpressed glycolate oxidase (GO) in rice at the whole plant level which resulted in an enhanced H_2_O_2_ concentration that triggered the stress tolerance response in transgenic plants (Cui et al., 2016). Contrary to Cui et al. (2016) our results indicate that at a relatively low salt concentration, the transgenic plants exhibited a significant increase in the seed germination index (Gi) as compared to WT plants, possibly due to the fact that the cyanobacterial genes do not produce H_2_O_2_ (Fig. 8). Transcriptomic profiling of GD plants also revealed the overexpression of oxidative stress tolerance related genes, which might have improved the stress tolerance response. Interestingly, among the transgenic plants, OX plants exhibited the highest tolerance as compared to GD and HD plants which may be attributed to oxalate metabolism by ODC (Fig. 8). However, this notion gives an indication that ODC expressing plants can be further evaluated for their potential to reduce the impact of other abiotic stresses as well. It has already been reported that the degradation of oxalate results in an improved stress tolerance specifically against fungal diseases and heavy metal stress (Donaldson et al., 2001; Wan et al., 2009; Xian et al., 2020). Ghosh et al. (2016) has also reported that oxalate reduces the oxidative burst during stress and regulates the defense mechanisms involved.

The contribution of different traits towards enhancing photosynthesis and biomass accumulation among transgenic plants can vary and few traits have more influence in causing overall variability than the others (Fig. 9). Dissection of traits as per their contribution in causing overall variability among the plants was carried out using PCA analysis (Afsar et al., 2020). In our study the PCA analysis identified *A*, g_s_, ΦPSII, ΦCO_2_, ETR, and E as significant traits positively associated with the enhancement of plant productivity (Table 1, Fig. 9A). Furthermore, the contribution of the traits identified through the PCA analysis has also shown that the contribution of genes to the improvement in biomass accumulation, followed the order GD > HD > OX (Fig. 9B).

The transcriptomic analysis provides an opportunity to identify other targets which can be manipulated to further enhance photosynthesis as well as biomass accumulation (Luo et al., 2019; Peng Yuan et al., 2020). Dalal et al. (2015) has reported 587 DEGs related to the amino acid biosynthesis pathway, cytochrome b, tryptophan biosynthesis, and superoxide dismutase, through transcriptomic profiling of *E. coli* GDH expressing transgenic plants. Similarly, in our study, transcriptomic profiling of GD plants not only showed that the genes related to photosynthesis were mainly overexpressed but also revealed the highest number of genes over expressed in response to abiotic stimulus (Figs. 10, 11). In the GD plants, ETR was significantly higher than WT plants, and the transcriptomic analysis also revealed an enhancement in genes expression related to the light harvesting complex (Lhc, a, and b) (Fig. 13) which further supports the role of GDH in enhancing photosynthesis. Concomitantly, overexpression of chlorophyll biosynthesis genes, such as magnesium chelatase (Mochizuki et al., 2001), putative dicarboxylate diiron (Kauss et al., 2012), chlorophyllide a oxygenase (Yang et al., 2016), and geranylgeranyl reductase (Lange & Ghassemian, 2003) was also observed in GD plants (Table 2). The mutants lacking the chlorophyllide a oxygenase gene in rice has been reported to impair the light harvesting complex, thereby affecting plant yield (Yang et al., 2016). Moreover, genes related to sugar and starch metabolism, such as cell wall invertases/Glycosyl hydrolase (Minic, 2008), β-glucosidase 1-6 (Singh, Verma & Kumar, 2016), trehalose phosphatase/synthase (Ponnu, Wahl & Schmid, 2011) and beta amylase 4 (Thalmann et al., 2019) were also upregulated (Table 2). Glycosyl hydrolases are involved in cell wall polysaccharide metabolism, modifications in sugars and secondary metabolites (Minic, 2008). β-amylases have diverse roles in plants, such as starch degradation in plastids, modulation of stomatal opening and transcriptional regulation in association with brassinosteroid signaling (Thalmann et al., 2019). In addition to these genes, glyoxylate metabolism related genes were also observed to be upregulated, including glyoxylate aminotransferase (Liepman et al., 2019), hydroxypyruvate reductase (Timm et al., 2011), serine acetyltransferase (Tavares et al., 2015), fumarase (Nunes-Nesi et al., 2007) glutamate dehydrogenase (Labboun et al., 2009), and 6-phosphogluconolactonase-1 (Xiong et al., 2009) (Table 2). Interestingly, these upregulated genes have a role in the biosynthesis of different metabolites involved in downstream reactions of the photorespiratory pathway involving glycine and glycerate metabolism, ammonia synthesis, and the production of reducing equivalents (Labboun et al., 2009; Xiong et al., 2009; Timm et al., 2011; Liepman et al., 2019). Consequently, the enhanced expression of these genes suggests an influence of GDH transformations on the regulation of the photorespiratory pathway. Furthermore, the transcriptomic analysis also revealed the overexpression of genes related to oxidative stress mitigation, such as glutathione S-Transferase (Gong et al., 2005), glutathione peroxidase 1 (Hasanuzzaman et al., 2017), and peroxisomal catalase (Mhamdi et al., 2010) (Table 2). These genes are thought to have a key role in reducing the oxidative burst during stress conditions (Gong et al., 2005; Mhamdi et al., 2010; Hasanuzzaman et al., 2017). Therefore, their upregulation resulting from the engineering of GDH indicates that they might have contributed to the increased salt stress tolerance of seed germination in GD plants.

## Conclusions

The current study revealed that the transformation of a single gene from the cyanobacterial glycolate decarboxylation pathway into *Arabidopsis thaliana* has the potential to enhance different photosynthetic, fluorescence and growth-related parameters. Elucidation of the underlying mechanism(s) involved *via* transcriptomic profiling of GD plants revealed an upregulation of the photorespiratory pathway, sugar and starch metabolism, and chlorophyll biosynthesis pathway genes. Based on these results the transformation of a single gene, has the potential to not only influence photorespiration but also modify genes in different metabolic pathways, with a consequent improvement in plant productivity. However, there is a need to carry out transcriptomic (HD and OX plants) and metabolomic profiling of GD, HD, and OX plants to enhance our understanding of the indirect effects of engineered cyanobacterial genes on improvements in plant biomass.

## Supplemental Information

10.7717/peerj.11860/supp-1 Supplemental Information 1 Raw data of photosynthesis analysis and biomass accumulation. Photosynthesis measurements of transgenic plants and biomass accumulation. It also contains raw data of seed weight, salt stress index and soulble sugars

10.7717/peerj.11860/supp-2 Supplemental Information 2 Measurement of Jc/Jo and Pr/Agross.

10.7717/peerj.11860/supp-3 Supplemental Information 3 Complete set of differentially regulated genes. The transcriptomic analysis revealed substantial set of genes which are differentially regulated in GD plants.

10.7717/peerj.11860/supp-4 Supplemental Information 4 Data Set of DEGS. Information of differentially up regulated genes in GD plants.

10.7717/peerj.11860/supp-5 Supplemental Information 5 Data set of DEGs. Data set containing information of down regulated genes in GD plants.

10.7717/peerj.11860/supp-6 Supplemental Information 6 Wild Type vs GD GO enrchment.

10.7717/peerj.11860/supp-7 Supplemental Information 7 Wild type vs GD KEGG enrichment.

10.7717/peerj.11860/supp-8 Supplemental Information 8 Wild type-1 FPKM.

10.7717/peerj.11860/supp-9 Supplemental Information 9 Wild type-2 FPKM.

10.7717/peerj.11860/supp-10 Supplemental Information 10 GD-1 FPKM.

10.7717/peerj.11860/supp-11 Supplemental Information 11 GD-2 FPKM.

## Additional Information and Declarations

### DNA Deposition

The following information was supplied regarding the deposition of DNA sequences: The sequences are available at NCBI: PRJNA705289.

### Data Availability

The following information was supplied regarding data availability: The raw data measurements, DEGS data, WT vs GD upregulated genes, GO enrichment, KEGG enrichment, FPKM of wild type and FPKM of GD are available in the Supplemental Files.
